# Trauma Activation Patients: Evidence for Routine Alcohol and Illicit Drug Screening

**DOI:** 10.1371/journal.pone.0047999

**Published:** 2012-10-19

**Authors:** C. Michael Dunham, Thomas J. Chirichella

**Affiliations:** Trauma/Critical Services, St. Elizabeth Health Center, Youngstown, Ohio, United States of America; California Pacific Medicial Center Research Institute, United States of America

## Abstract

**Background:**

Statistics from the National Trauma Data Bank imply that discretionary blood alcohol and urine drug testing is common. However, there is little evidence to determine which patients are appropriate for routine testing, based on information available at trauma center arrival. In 2002, Langdorf reported alcohol and illicit drug rates in Trauma Activation Patients.

**Methodology/Principal Findings:**

This is a retrospective investigation of alcohol and illicit drug rates in consecutive St. Elizabeth Health Center (SEHC) trauma patients. SEHC Trauma Activation Patients are compared with the Langdorf Activation Patients and with the SEHC Trauma Nonactivation Patients. Minimum Rates are positive tests divided by total patients (tested and not tested). **Activation patients:** The minimum alcohol rates were: SEHC 23.1%, Langdorf 28.2%, combined 24.8%. The minimum illicit drug rates were: SEHC 15.7%, Langdorf 23.5, combined 18.3%. The minimum alcohol and/or illicit drug rates were: SEHC 33.4%, Langdorf 41.8%, combined 36.2%. **Nonactivation patients:** The SEHC minimum alcohol rate was 4.7% and the minimum illicit drug rate was 6.0%.

**Conclusions:**

Alcohol and illicit drug rates were significantly greater for Trauma Activation Patients, when compared to Nonactivation Patients. At minimum, Trauma Activation Patients are likely to have a 1-in-3 positive test for alcohol and/or an illicit drug. This substantial rate suggests that Trauma Activation Patients, a readily discernible group at trauma center arrival, are appropriate for routine alcohol and illicit drug testing. However, discretionary testing is more reasonable for Trauma Nonactivation Patients, because minimum rates are low.

## Introduction

It is clear that American trauma leadership endorses alcohol and drug testing. The National Trauma Data Bank (NTDB), sponsored by the American College of Surgeons Committee on Trauma, is the largest database of trauma center admissions in the United States [1]. The inclusion of alcohol and drug results in the NTDB is an indication that the Committee supports alcohol and drug testing. Further, the submission of alcohol and drug results to the NTDB from several hundred trauma centers represents endorsement of alcohol and drug testing by trauma directors. Peer-reviewed publications of alcohol and drug results from the NTDB represent additional evidence that alcohol and drug testing is important [2], [3]. Two statements from the Advanced Trauma Life Support Student Course Manual support the importance of considering the role that alcohol and drugs may play in trauma [4]. The first relevant statement is on page 271: “Abuse of alcohol and/or other drugs is common to all forms of trauma and is particularly important to identify”. The second germane comment is on page 281: “Abuse of alcohol and other drugs is an example of a contributory factor that is likely to be pervasive regardless of whether the trauma is blunt or penetrating”.

Discretionary, non-universal, alcohol and drug testing appears to be the typical practice. A review of results from the NTDB, after excluding not-applicable patients, shows that only 39% underwent alcohol testing and 26% had urine toxicology testing [1]. Although alcohol and drugs may play a seminal role in trauma, there are no clear directives as to who should or should not be tested.

One problem with alcohol and drug reporting in trauma patients is that the description of the parent trauma population is commonly nebulous. Examples include “3,312 trauma patients came to our facility” [5]; “subjects were patients who were admitted to the hospital for at least 48 hours following a traumatic injury” [6]; and “all trauma patients 12 years of age or older treated in the LBMMC's Emergency Department” [7]. Because it is unclear why some were tested and others were not, potential selection biases may limit generalized inferences.

Several investigators support a notion of select urine toxicology testing in trauma patients [5], [8], [9]. Specifically, Vitale proposed that studies to identify clearly delineated trauma risk groups for illicit drugs are needed [9]. Investigators emphasize the potential value of identifying patients with alcohol and illicit drug use for the purpose of intervention and prevention of subsequent trauma [9], [10].

Although the positive alcohol rate in a study by Blondell was 29.3%, [6] the NTDB report provided a higher incidence, 39.0% [1]. The NTDB rate represents a 33% higher value than that reported by Blondell. Additional literature discrepancy is seen when comparing the illicit drug rates reported by Langdorf [8] and Buchfuhrer [7]. While the positive illicit drug rate was 27.8% in the study by Langdorf, Buchfuhrer reported a 60% higher incidence (45.7%). The literature alcohol and illicit drug rate discrepancies and the lack of clear indications for which patients should undergo testing imply that additional insight is needed. We hypothesize that trauma activation patients are likely to have higher rates of alcohol and illicit drug detection, when compared to nonactivation trauma center admissions.

## Materials and Methods

### Ethics Statement

The St. Elizabeth Health Center (SEHC) Institutional Review Board waived the need for informed consent. The SEHC Institutional Review Board approved the study and the data were deidentified and analyzed anonymously.

This is a retrospective study of consecutive patients evaluated by the trauma services at SEHC, an urban Level I trauma center, from June through the end of August in 2010. SEHC patients consist of Trauma Activation Patients and Trauma Nonactivation Patients. SEHC alcohol and illicit drug rates in consecutive Trauma Activation Patients were computed and compared with the Langdorf Trauma Activation Patients [8]. The Langdorf publication, the only other study of consecutive Trauma Activation Patients, was performed in a comparable urban Level I trauma center and had a high alcohol and drug toxicology-testing rate (85%). Alcohol and illicit drug results for the SEHC Trauma Activation Patients were then compared with the SEHC Trauma Nonactivation Patients. Alcohol and illicit drug rates for all SEHC Trauma Center Admissions were then compared with results for the NTDB Trauma Center Admissions [1].

SEHC Trauma Center Admissions consisted of the combined SEHC Trauma Activation Patients and the SEHC Trauma Nonactivation Patients. SEHC Trauma Activation Patients included those with a Trauma Team status or Trauma Alert status. Trauma Team Activations were employed for physiologic or anatomic indicators and Trauma Alert Activations were implemented for high-risk blunt trauma mechanisms. Trauma Activation Patients were evaluated by the trauma services immediately upon emergency department arrival. Trauma Nonactivation Patients were other patients initially evaluated by the emergency department physician with a request for consultation and admission to the Trauma Service.

Violent mechanisms of injury included gunshot wounds, stab wounds, and interpersonal blunt trauma assaults. For the SEHC patients, clinical traits and outcomes were obtained from the trauma registry which participates in the NTDB. Blood alcohol results were from the Trauma Registry and urine toxicology results emanated from the medical records. Blood alcohol was considered positive when any measurable level was present. Using Siemen's Advia 1800 instrumentation, blood alcohol concentrations were computed based on alcohol dehydrogenase and nicotinamide adenine dinucleotide reactions. When a patient's urine drug screen was positive for an amphetamine, cannabinoid, cocaine, or phencyclidine, an illicit drug was considered as present. Using Siemen's Advia 1800 instrumentation, illicit drugs were initially identified by an enzyme multiplied immunoassay technique. Positive screens were confirmed using gas chromatography mass spectrometry for cocaine and thin layer chromatography for the other illicit drugs.

Trauma activation criteria are compared with the American College of Surgeons Committee on Trauma, Trauma Center Triage criteria [11]. Arrival from scene is defined as patients transported directly from the site of injury to the Emergency Department and immediately evaluated by the Trauma Service. The Glasgow Coma Scale (GCS) was documented on emergency department arrival. Age of each patient was obtained from the medical record, and the Injury Severity Score (ISS) was obtained from the Trauma Registry.

### Statistics

A Minimum Alcohol Rate and Minimum Illicit Drug Rate were computed as the number of positive tests divided by the number of total patients in the cohort (tested and not tested). This computation produces a minimum alcohol or illicit drug positive rate for the parent population. A Tested Alcohol Rate and Tested Illicit Drug Rate were calculated by dividing the number of patients with a positive alcohol or toxicology screen by the number of patients in the cohort who were tested. Tested Rates exclude those not tested from the trauma cohort denominator, thus creating a potential sampling bias that can produce a distorted alcohol or illicit drug positive rate for the parent population.

SAS System for Windows, release 9.2 (SAS Institute Inc., Cary, NC, USA) was used to perform the statistical analysis. Interval data are presented as the mean and standard deviation. Nonparametric data are presented as the mean and range. We considered P<0.05 to represent statistical significance.

## Results

From June through August 2010, 338 consecutive SEHC Trauma Activation Patients were evaluated. Injury traits and outcomes are in Table 1. The GCS mean was relatively high: GCS 3–8 n = 43 (12.7%), GCS 9–12 n = 19 (5.6%), and GCS 13–15 n = 276 (81.7%). Alcohol and illicit drug testing and positive rates for SEHC Trauma Activation Patients are in Table 2. Amphetamines were present in five patients, cannabinoid in 40, cocaine in 16, and phencyclidine was not found in any patient. These 61 positive tests occurred in a total of 53 patients. A comparison of blood alcohol and illicit drug results for the SEHC and Langdorf Trauma Activation Patients studies is displayed in Table 2. Because each Trauma Activation Patient could potentially have two tests (blood alcohol and urine toxicology), the testing rate for alcohol and/or an illicit drug (percent tested) is based on the number of tests performed relative to the potential number. For example, the 338 patients in the SEHC study could potentially have 676 tests. The testing rate for the SEHC Trauma Activation Patients study was 71.0% (480/676). The percent of Trauma Activation Patients having both blood alcohol and urine toxicology testing is 84.7% (n = 144) in the Langdorf study and 49.7% (n = 168) in the SEHC study. The Trauma Activation Patients positive-alcohol and/or illicit drug Tested Rate for the Langdorf study (49.3% [71/144]) is similar to that of the SEHC study (52.4% [88/168]; P = 0.59). Trauma Activation Patients inclusion criteria for the SEHC and Langdorf studies were similar, as displayed in Table 3.

**Table 1:** SEHC Trauma Activation Patients traits and outcomes (n = 338).

**Violent Mechanism**	47	13.9%
**Arrival from Scene**	283	83.7%
**Trauma Team Status**	99	29.3%
**Age (years)**	40.5±20.6	
**Glasgow Coma Score**	13.2 (3–15)	
**Injury Severity Score**	10.6 (1–57)	
**Died**	20	5.9%
**Length of Stay (days)**	5.0±8.1	

SEHC, St. Elizabeth Health Center; Violent mechanism: gunshot wound, stab wound, or assault.

**Table 2:** SEHC and Langdorf Trauma Activation Patients alcohol and illicit drug minimum and tested rates.

Alcohol Rates:						
Study	# Pts.	# Tested	% Tested	# (+)	Minimum Rate+	Tested Rate++
SEHC	338	295	87.3%	78	23.1%	26.4%
Langdorf	170	144	84.7%	48	28.2%	33.3%
Both	508	439	86.4%	126	**24.8%**	**28.7%**
**Illicit Drug Rates:**						
SEHC	338	185	54.7%	53	15.7%	28.6%
Langdorf	170	144	84.7%	40	23.5%	27.8%
Both	508	329	64.8%	93	**18.3%**	**28.3%**
**Alcohol and/or Illicit Drug Rates:**						
SEHC	338	480/676	71.0%	113	33.4%	
Langdorf	170	288/340	84.7%	71	41.8%	
Both	508	768/1016	75.6%	184	**36.2%**	

SEHC, St. Elizabeth Health Center; # Pts., number of patients; # Tested, number of patients tested; % Tested, percent of patients tested; # (+), number of positive tests.

+ **Minimum Rate** is number of positive tests divided by total patients, tested and not tested.

++ **Tested Rate** is number of positive tests divided by number of patients tested.

**Table 3:** Trauma Activation Patients inclusion criteria.

ACS Trauma Center Triage	Langdorf	SEHC
Glasgow Coma Score <14	Yes	yes
systolic blood pressure <90	Yes	yes
respiratory rate <10 or >29	Yes	yes
penetrating injury head, neck, torso, or proximal extremity	Yes	yes
flail chest	Yes	yes
≥2 proximal long-bone fractures	Yes	yes
crushed, degloved, or mangled extremity	No	yes
amputation proximal to wrist or ankle	Yes	yes
suspected pelvic fracture	Yes	yes
open or depressed skull fracture	No	yes
Paralysis	Yes	yes
trauma with burns	Yes	yes
high-risk blunt trauma mechanism	Yes	yes

ACS, American College of Surgeons; SEHC, St. Elizabeth Health Center.

Comparisons of Trauma Activation Patients and Trauma Nonactivation Patients in the SEHC study are in Table 4. SEHC Trauma Activation Patients, in comparison to SEHC Trauma Nonactivation Patients, had greater violent mechanisms, more motor vehicular crashes, fewer falls, lower age, lower GCS, and higher injury severity. The alcohol Minimum Rate was significantly greater for Trauma Activation Patients, when compared to Trauma Nonactivation Patients (OR 6.1; P<0.0001). The illicit drug Minimum Rate was significantly higher for Trauma Activation Patients, when compared to Trauma Nonactivation Patients (OR 2.9; P = 0.0003).

**Table 4:** Comparison of SEHC Trauma Activation Patients and SEHC Trauma Nonactivation Patients, minimum alcohol and illicit drug rates.

	Activations		Non Activations		P-value
	n = 338		n = 234		
**Mechanism of Injury**					
violent	47	13.9%	16	6.8%	0.008
fall	82	24.3%	156	66.7%	0.0001
motor vehicular crash	93	27.5%	29	12.4%	0.0001
**Age (years)**	41±21		59±26		0.0001
**Glasgow Coma** **Score**	13 (3–15)		15 (10–15)		0.0001
**Injury Severity** **Score**	11 (1–57)		8 (2–26)		0.0001
**Died**	20	5.9%	1	0.4%	0.001
**Length of Stay** **(days)**	5±8		4±4		0.0001
**Percent Tested**					
blood alcohol	295	87.3%	43	18.4%	0.0001
urine drug	185	54.7%	47	20.1%	0.0001
**Blood Alcohol** **Positive**	78	**23.1%**	11	**4.7%**	0.0001
**Illicit Drug** **Positive**	53	**15.7%**	14	**6.0%**	0.0003

SEHC, St. Elizabeth Health Center; Violent mechanism: gunshot wound, stab wound, or assault.

**Minimum Rate** is number of positive tests divided by total patients, tested and not tested.

SEHC Trauma Center Admissions (combined SEHC Trauma Activation Patients and SEHC Trauma Nonactivation Patients) and NTDB Trauma Center Admissions results are in Table 5. The alcohol Minimum Rates for SEHC and NTDB Trauma Center Admissions were similar; as were the illicit drug Minimum Rates. However, alcohol Tested Rates for the two investigations were different; as were the illicit drug Tested Rates. The proportion of patients undergoing testing for alcohol and illicit drugs was ≤60%, for both SEHC and NTDB Trauma Center Admissions. Alcohol and illicit drug testing rates are substantially lower for the NTDB, relative to SEHC.

**Table 5:** Comparison of SEHC Trauma Center Admissions with NTDB Trauma Center Admissions, alcohol and illicit drug rates.

Alcohol Rates:						
Study	# Pts.	# Tested	% Tested	# (+)	Minimum Rate+	Tested Rate++
SEHC	572	338	59.1%	89	15.6%	26.3%
NTDB 2010+++	650,858	252,781	38.8%	98,517	15.1%	39.0%
**Illicit Drug Rates:**						
SEHC	572	232	40.6%	67	11.7%	28.9%
NTDB 2010++++	590,221	155,039	26.3%	65,247	11.1%	42.1%

SEHC, St. Elizabeth Health Center; NTDB, National Trauma Data Bank.

SEHC Trauma Center Admissions are SEHC Trauma Activation Patients combined with SEHC Trauma Nonactivation Patients.

# Pts., number of patients; # Tested, number of patients tested; % Tested, percent of patients tested; # (+), number of positive tests.

+ **Minimum Rate** is number of positive tests divided by total patients, tested and not tested.

++ **Tested Rate** is number of positive tests divided by number of patients tested.

+++ Data from Table 13 in 2010 Report excludes Not Applicable patients.

++++ Data from Table 14 in 2010 Report excludes Not Applicable patients.

## Discussion

Commonly, the literature describes trauma-associated alcohol and drug rates for patients “undergoing alcohol and/or urine toxicology testing”. However, the patient traits of those with and without testing, i.e., selection biases, are typically not elucidated. When reviewing the trauma literature for alcohol and illicit drug rate results, potential sampling bias and errors need consideration. Specifically, determine whether a) the traits of the trauma cohort are clear, b) all patients were tested, and c) all patients were included in the analysis. A Minimum Alcohol or Illicit Drug Rate is computable if the trauma patient cohort undergoes select alcohol or illicit drug testing, yet the analysis includes all patients (tested and not tested) in the trauma cohort denominator. This computation produces a minimum, lowest possible, alcohol or illicit drug positive rate for the parent population.

### Trauma Activation Patients

The Langdorf study and a subset of SEHC patients provide a retrospective review of consecutive Trauma Activation Patients from two Level I trauma centers. Based on these investigations, Trauma Activation Patients are likely to have a 1-in-4 positive test for blood alcohol, a 1-in-5 positive test for an illicit drug, and a 1-in-3 positive test for alcohol and/or an illicit drug (Table 2).

Of the patients positive for an illicit drug in the SEHC study of Trauma Activation Patients, cannabinoid and cocaine were relatively common, amphetamines were infrequent, and phencyclidine was nonexistent. Other trauma studies have also shown that cocaine [5], [7], [8], [9], [12], [13] and cannabinoid [5], [7], [8], [9], [13] detection are relatively common. In contrast to the SEHC study of Trauma Activations Patients, Langdorf found that amphetamine was a fairly, frequent finding [8]. However, he also noted that phencyclidine was uncommon [8].

Alcohol testing rates were approximately 85% in the Langdorf and SEHC studies of Trauma Activation Patients (Table 2). Note that the alcohol Minimum Rate is similar to the Tested Rate for each study, indicating that when testing rates are high, Minimum and Tested Rates tend to be comparable. It is important to consider that the Minimum Rates of the two studies were similar, as were the Tested Rates. These findings enhance the likelihood that the alcohol-positive Minimum and Tested Rates from these studies are a reliable representation for other Trauma Activation Patients. It makes sense that when the testing rate is high, the Minimum and Tested Rates will likely approach a rate that is accurate and non-biased.

The illicit drug-testing rate was approximately 85% in the Langdorf study of Trauma Activation Patients (Table 2). Similar to the alcohol results with a high testing rate, the illicit drug Minimum and Tested Rates were analogous. In contrast, the illicit drug-testing rate in the SEHC study of Trauma Activation Patients was lower. Trauma surgeon bias regarding the value of testing and the presence or absence of a urinary bladder catheter may have played a role. However, these factors are uncertain. Apropos, the Minimum Rate was less than the Tested Rate and the Minimum Rate was lower in comparison to the Langdorf study. This finding exemplifies that the illicit drug-testing rate has an influence on the Minimum Rate.

### Trauma Activation Patients Comparison with Trauma Nonactivation Patients

A comparison of Trauma Activation Patients and Trauma Nonactivation Patients in the SEHC study is elucidating (Table 4). Mechanism of injury, age, admission Glasgow Coma Score, and Injury Severity Score are significantly different for the two groups. Of importance, the blood alcohol and illicit drug testing-rates and the Minimum Rates are substantially lower for the Trauma Nonactivation Patients. These observations suggest that discretionary alcohol and drug testing for Trauma Nonactivation Patients is reasonable.

### Trauma Center Admissions (Trauma Activation and Nonactivation Patients)

It is clear that United States trauma leadership embrace blood alcohol and urine drug testing. The inclusion of alcohol and drug testing in the NTDB and the submission of data by trauma directors support this notion. Discretionary (non-universal) blood alcohol and urine toxicology testing is a common practice in trauma centers. The blood alcohol-testing rate was 60% for the Trauma Center Admissions (combined Trauma Activation Patients and Trauma Nonactivation Patients) in the SEHC study (Table 5). Other studies have reported alcohol-testing rates of 40 [1] and 75% [6], [7]. Additional evidence for discretionary testing is the 40% urine toxicology-testing rate in the SEHC study of Trauma Center Admissions (combined trauma activation and Nonactivation patients) (Table 5). The NTDB 2010 report of Trauma Center Admissions (combined Trauma Activation and Nonactivation Patients) indicates an illicit drug testing rate of only 25% [1].

In the two studies of Trauma Center Admissions (combined Trauma Activation and Nonactivation Patients), the testing rate for alcohol was lower in the NTDB study (38.8%) when compared to the SEHC study (59.1%). However; of those tested, more NTDB Trauma Center Admissions patients were positive (39.0%) than those in the SEHC study (26.3%). This demonstrates how sampling bias might influence the alcohol-positive Tested Rate. The Minimum Rates for alcohol in the two Trauma Center Admissions (SEHC and NTDB) studies were lower in comparison to those for the two Trauma Activation Patients cohorts (SEHC and Langdorf).

An examination of the illicit drug rates in the two Trauma Center Admissions studies reveals comparable issues. The testing rate was lower in the NTDB (26.3%), when compared to the SEHC study of Trauma Center Admissions (40.6%). However; of those tested, the NTDB positive rate was higher (42.1%), when compared to the SEHC study (28.9%). This suggests that there is likely variance in patient selection and exclusion between the two investigations. This finding also implies that selection biases can influence illicit drug Tested Rates. It is germane that London, following an analysis of the NTDB, demonstrated that trauma patient drug testing is decreasing [2]. Of relevance, several investigators support a notion of select urine toxicology testing in trauma patients [5], [8], [9].

The comparison of SEHC Trauma Activation Patients with SEHC Trauma Nonactivation patients shows higher alcohol and illicit drug Minimum Rates for Activation Patients. Of note, SEHC Activation Patients, in comparison to Nonactivation Patients, had greater violent mechanisms, more motor vehicular crashes, fewer falls, lower age, lower GCS, and higher injury severity (Table 4). Although these traits might be considered as potential risk factors for positive alcohol and illicit drug tests, we did not perform a risk factor analysis. Other investigators have described host risks for positive alcohol and illicit drug tests. Blondell showed that increased alcohol and cocaine positive results were associated with a violent mechanism of injury [13]. In an earlier publication, Blondell noted that alcohol positive rates were related to age ≤40 years old [6]. Also, Vitale demonstrated an association between illicit drugs and age 20–40 and violence [9].

Langdorf proposed a multifaceted set of rules, based on time of injury, mechanism of injury, and patient age, as to when toxicology screening should or should not be performed [8]. Such a complex policy can be difficult to reliably implement when confronted with the challenges of evaluating and managing a critically injured patient. Further, a risk factor analysis only indicates that a particular trait is associated with an increased event. It does not necessarily imply that the alcohol or illicit drug rate would not be substantial in the lower risk cohort. Our SEHC study findings indicate that the positive rate of alcohol and/or illicit drug tests in Trauma Activation Patients is substantial. The results also show that trauma activation, relative to nonactivation, is a risk factor for alcohol and illicit drug positivity. A policy of routine alcohol and illicit drug testing in Trauma Activation Patients represents a noncomplex strategy, which would likely foster institutional compliance.

### Limitations

Although this is a retrospective study, it is an analysis of consecutive trauma patients who either did or did not undergo blood alcohol testing or urine drug screening. We consider the trauma registry to be a reliable database. However, data accuracy and quality from a retrospective database source is lower, when compared to a prospective, dedicated database. The determination of patients consuming a narcotic or sedative prior to their trauma event may have been elucidating. Our study uses discretionary, non-universal, alcohol and urine drug testing. Thus, an accurate rate for the parent population is uncertain.

### Conclusions

Alcohol and illicit drug minimum rates are significantly greater for Trauma Activation Patients, when compared to Trauma Nonactivation Patients. Trauma Activation Patients are likely to have, at least, a 1-in-4 positive test for blood alcohol, a 1-in-5 positive test for an illicit drug, and a 1-in-3 positive test for alcohol and/or illicit drug. Optional alcohol and urine toxicology testing is a common practice in American trauma centers. However, guidelines for testing or not testing, based on information available at trauma center arrival, are not established. The data in this report indicate that Trauma Activation Patients have substantial exposure to alcohol and illicit drugs. This suggests that Trauma Activation Patients, a readily discernible group at trauma center arrival, are appropriate for routine alcohol and illicit drug testing. The relatively low alcohol and illicit drug rates in Trauma Nonactivation Patients imply that discretionary testing is more reasonable.
